# Learning from long‐term adolescent and young adult (AYA) cancer survivors regarding their age‐specific care needs to improve current AYA care programs

**DOI:** 10.1002/cam4.6001

**Published:** 2023-04-29

**Authors:** Silvie H. M. Janssen, Carla Vlooswijk, Eveliene Manten‐Horst, Sophia H. E. Sleeman, Rhodé M. Bijlsma, Suzanne E. J. Kaal, Jan Martijn Kerst, Jacqueline M. Tromp, Monique E. M. M. Bos, Tom van der Hulle, Roy I. Lalisang, Janine Nuver, Mathilde C. M. Kouwenhoven, Winette T. A. van der Graaf, Olga Husson

**Affiliations:** ^1^ Department of Psychosocial Research and Epidemiology Netherlands Cancer Institute Amsterdam The Netherlands; ^2^ Department of Medical Oncology Netherlands Cancer Institute – Antoni van Leeuwenhoek Amsterdam The Netherlands; ^3^ Research and Development Netherlands Comprehensive Cancer Organization Utrecht The Netherlands; ^4^ Dutch AYA ‘Young & Cancer’ Care Network Utrecht The Netherlands; ^5^ Department of Medical Oncology University Medical Center Utrecht The Netherlands; ^6^ Department of Medical Oncology Radboud University Medical Center Nijmegen The Netherlands; ^7^ Department of Medical Oncology Amsterdam University Medical Centers Amsterdam The Netherlands; ^8^ Department of Medical Oncology Erasmus MC Cancer Institute, Erasmus University Medical Center Rotterdam The Netherlands; ^9^ Department of Medical Oncology Leiden University Medical Center Leiden The Netherlands; ^10^ Department of Internal Medicine GROW‐School of Oncology and Reproduction, Maastricht UMC+ Comprehensive Cancer Center Maastricht The Netherlands; ^11^ Department of Medical Oncology University Medical Center Groningen Groningen The Netherlands; ^12^ Department of Neurology Amsterdam UMC, Amsterdam University Medical Centers, Location VUmc Amsterdam The Netherlands; ^13^ Department of Surgical Oncology Erasmus MC Cancer Institute, Erasmus University Medical Center Rotterdam The Netherlands

**Keywords:** adolescents and young adults, age‐specific care needs, AYAs, cancer, population‐based data, survivorship

## Abstract

**Background:**

Despite growing (inter)national awareness and appreciation, age‐specific care is still not always self‐evident and accepted as standard of care for adolescent and young adult (AYA) cancer patients. It is unknown whether long‐term AYA cancer survivors have missed age‐specific care, and if so, which survivors missed it and regarding which topics.

**Methods:**

The Netherlands Cancer Registry (NCR) identified all long‐term AYA cancer survivors (aged 18–39 years at initial cancer diagnosis, 5–20 years past diagnosis) in the Netherlands, who were invited to participate in a population‐based, observational, cross‐sectional questionnaire study (SURVAYA study), including questions on care needs.

**Results:**

In total, 3.989 AYAs participated (35.3% response rate). One‐third of them had a need for age‐specific care (33.5%), 41.2% had no need and 25.3% did not know whether they had a need. Those who had a need for age‐specific care were significantly more often female, higher educated, diagnosed at a younger age, and treated with chemotherapy, radiotherapy or hormone therapy. Most frequent topics were disease and treatment (29.7%), emotions (24.1%), friends (22.6%), family and children (15.6%), fertility and pregnancy (14.8%), work and reintegration (10.5%), care not tailored (13.8%), and overarching care and life (27.7%). Palliative care (0.0%), spirituality (0.2%), death (0.7%), complementary care (0.7%), and late effects (1.3%) were mentioned least.

**Conclusions:**

A substantial proportion of long‐term AYA cancer survivors showed a need for age‐specific care, varying by sociodemographic and clinical factors, on a wide variety of topics, which could be targeted to improve current AYA care services.

## Graphical abstract

Based on population‐based, observational, cross‐sectional questionnaire study data, a substantial proportion of long‐term (5–20 years post‐diagnosis) adolescent and young adult (AYA) cancer survivors (18–39 years at initial cancer diagnosis) show a need for age‐specific care, varying by sociodemographic and clinical factors, on a wide variety of topics, which could be targeted to improve current AYA care services.

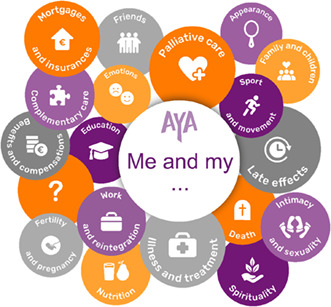

## 1 INTRODUCTION

Over the past decades, the recognition of the distinctive challenges in care provision to the adolescent and young adult (AYA) cancer population has increased in several countries.^1^ AYAs are defined as those aged 15–39 years at initial cancer diagnosis according to the United States' National Cancer Institute (NCI). However, the NCI also states that this age range can be flexibly applied, depending on the type of healthcare delivery system for example.^2^ Although the AYA age group spans both the traditional pediatric and adult healthcare systems, it does not fit well into either with respect to meeting their clinical needs.^1^, ^3^, ^4^, ^5^ Based on international initiatives, like Bleyer and colleagues' research and the landmark report of the National Cancer Institute/LIVESTRONG's Progress Review Group, highlighting the lack of improvement in AYAs' survival rates (compared to childhood and adult cancer survivors), recommendations to improve care for and outcomes of AYAs were formulated.^6^, ^7^ Over time, these initiatives have been further supported by the start of working groups, task forces, (inter)national AYA organizations and committees, and a journal solely devoted to AYAs with cancer.^1^, ^8^, ^9^, ^10^, ^11^, ^12^ In addition, national AYA‐specific care programs have been developed in numerous countries, based on the local needs and available resources.^5^, ^8^, ^11^

In the Netherlands, age‐specific care for AYAs was initiated by the national AYA “Young & Cancer” Care Network in 2016. It was kick started by a manifest in which AYAs and healthcare professionals (HCPs) raised their voice, after which the foundation was laid for age‐specific care based on the input of AYAs and HCPs.^13^, ^14^ The self‐identified needs of this population include, among others, dealing with challenges in body image, sexuality, relationships with family and friends (including partners), fertility, education and employment, and financial stressors.^14^ Currently, more than half of the Dutch hospitals are officially providing age‐specific care and this number is still increasing, leading to improved national coverage.^15^ The network is aimed at a constant development of care, education and research, which has led to an anamnesis tool built in the electronic patient files, quality standards, care pathways for the implementation of care, and a training plan for HCPs, including education, intervision, and e‐learning modules. The care pathways and HCPs' expertise, including specialists, nurses, and paramedics, remain up‐to‐date through the integration of research.^12^, ^14^, ^15^, ^16^

Despite growing (inter)national awareness and appreciation, age‐specific care was and still is not always self‐evident and accepted as standard of care for this population.^5^, ^12^, ^17^, ^18^ This may lead to unmet (supportive care) needs and poor outcomes (e.g., regarding fertility, education and work, and quality of life). The systematic reviews of Bibby et al. and Galán et al. showed that commonly reported needs of AYAs relate to communication and information delivery, fertility, psychological support, social support, and contact with other AYA cancer patients.^19^, ^20^ Important to note is that, up till now, most studies have focused on reporting the (unmet) needs of AYAs during the first months or years following diagnosis only.^19^, ^21^, ^22^

Most AYAs survive their initial cancer (>80%) and many long‐term AYA cancer survivors, who were diagnosed before the recent developments in age‐specific care, may have never had the opportunity to receive this type of care. Looking back now, it is unknown if these long‐term survivors have had a need for age‐specific care at all, and if so, regarding which topics they needed it. It is expected that some of them have had a need for age‐specific care, but differences may exist based on clinical and sociodemographic characteristics. Using the AYA cancer survivor population's experiences as input, current age‐specific care for AYAs can be re‐evaluated, refined, and better attuned to future AYA cancer patients’ needs and wishes. Therefore, the aims of this retrospective, population‐based, observational, cross‐sectional cohort study are to examine (1) whether there has been a need for age‐specific care among long‐term AYA cancer survivors in the Netherlands; (2) which AYA cancer survivors have had a need for age‐specific care (based on sociodemographic and clinical characteristics); and (3) regarding which topics there has been a need for age‐specific care.

## 2 METHODS

### 2.1 Data collection

This paper presents a secondary data analysis of the SURVAYA study data. The main aim of the retrospective, observational cohort study was to examine the prevalence, risk factors, and mechanisms of impaired health outcomes among a population‐based sample of AYA cancer survivors (Clinical trials registration: NCT05379387).^23^ A detailed description of the methods used in the SURVAYA study has been reported previously.^24^ In short, all AYA cancer survivors diagnosed with their first invasive tumor between 1999 and 2015, at the age of 18–39 years, in one of the nine participating cancer centers, were selected from the Netherlands Cancer Registry (NCR). The population‐based NCR collects disease‐ and treatment‐specific data of all cancer patients in the Netherlands since 1989.^25^ In order to check whether AYAs were still alive at the moment of invitation and to obtain up‐to‐date addresses, records were linked to and checked with the Dutch municipal records database.

All eligible AYAs received an invitation for participation in the SURVAYA study from their (former) medical specialist via PROFILES (Patient Reported Outcomes Following Initial treatment and Long‐term Evaluation of Survivorship). PROFILES is a registry to study the impact of cancer (treatment) from a population‐based cohort of survivors.^26^ After signing the informed consent form, the questionnaire could be completed either online or on paper.^24^ After completion, the questionnaire data were linked to the clinical data of the NCR to finalize the dataset. The Institutional Review Board of the Antoni van Leeuwenhoek—Netherlands Cancer Institute approved this study (IRBd18122) and the NCR approved linkage, access and utilization of their clinical data.

### 2.2 Measures

#### 2.2.1 Sociodemographic characteristics

Sex and age at diagnosis were available from the NCR. Marital status, educational level (highest level achieved), and living status were self‐reported by the participants.

#### 2.2.2 Clinical characteristics

Time since diagnosis, type of cancer (classified according to the Third International Classification of Diseases for Oncology (ICDO‐3)), type of primary treatment and tumor stage (classified according to TNM or Ann Arbor Code) were available from the NCR.

#### 2.2.3 Need for age‐specific care

Participants were asked to answer the following question: “Would you have needed age‐specific (AYA) care due to your cancer”? with answering options “yes,” “no,” or “do not know.” In addition, they had the option to specify their need(s) in an open text box.

### 2.3 Data analysis

**FIGURE 1:**
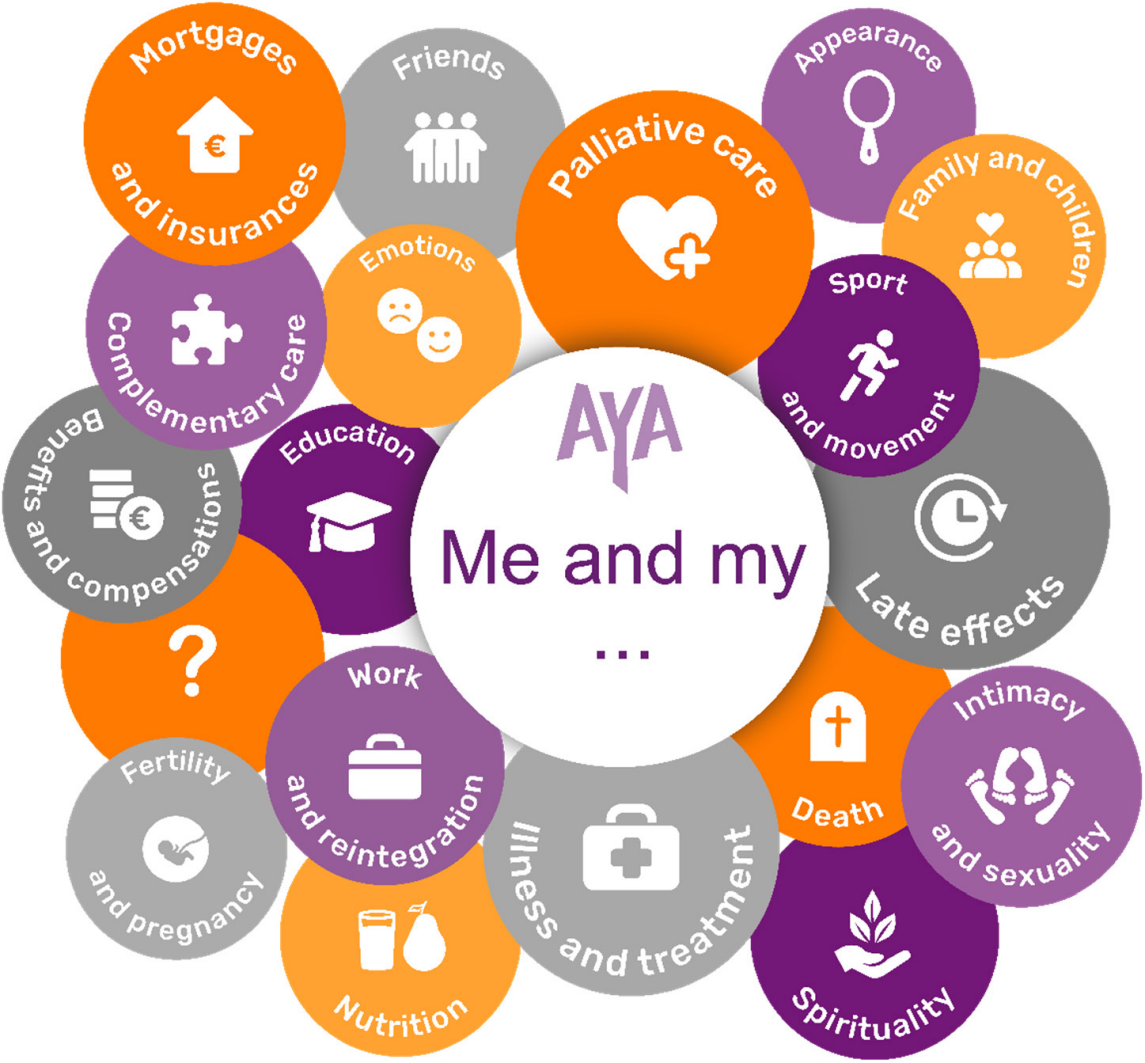
Dutch AYA “Young & Cancer” Care Network's AYA anamnesis for clinical practice. *Note*: the AYA anamnesis tool for clinical practice is used to facilitate the discussion between the HCP and the AYA during the consultation. Each topic represents a need with questions and possible interventions.

Answers to the question “Please specify your age‐specific (AYA) needs” were categorized according to a framework based on the Dutch AYA “Young & Cancer” Care Network's AYA anamnesis for clinical practice (Figure 1).^14^ Two researchers (S.H.M.J. and C.V.) independently categorized all open text responses using an inductive approach, thus allowing for new (sub) topics to arise based on the data. Multiple topics could be selected per open text response. A third researcher (O.H.) was consulted to discuss deviations and come to an agreement. When consensus was reached, S.H.M.J. finalized the categorization.

Statistical analyses were performed using IBM SPSS Statistics version 25 (SPSS Inc.). Two‐sided *p*‐values of <0.05 were considered statistically significant. Descriptive statistics were used to characterize the study population. Chi‐square and ANOVA tests (including Games–Howell post hoc test) were carried out to make comparisons between those with, without and who do not know whether they have had a need for age‐specific care, on sociodemographic and clinical characteristics.

## 3 RESULTS

The NCR identified 17.098 AYA cancer survivors, of which 11.296 were invited for participation. Reasons to exclude patients from participation were as follows: missing up‐to‐date information on vital status and/or address, not having a histological diagnosis, having a very good prognosis or extreme rare tumor type at this age (i.e., might have been unclear to the AYA that one is diagnosed with cancer) as defined by Vlooswijk et al.,^24^ and not having permission of the hospital to invite the patient for study participation, that is, only inviting AYAs with certain tumor types. Eventually, 35.5% of the invited AYAs completed the questionnaire, leading to an observational cohort of 4.010 AYA cancer survivors. Of these, 21 AYAs did not complete the question on whether they have had a need for age‐specific care and were therefore excluded from further analysis (final cohort N = 3.989).

### 3.1 AYA cancer survivors' need for age‐specific care

Table 1 shows the characteristics of the included AYA cancer survivors classified according to their reported need for age‐specific care. The total study population is described in Table A1. The participants were on average 12.4 years post‐diagnosis, and the most common cancer types included breast cancer (23.5%), germ cell tumor (17.3%), and lymphoid hematological malignancy (14.7%) (Table A1). Of all AYAs, 33.5% expressed a need for age‐specific care, while 41.2% expressed no need and 25.3% did not know whether they have had a need for age‐specific care (Table 1).

**TABLE 1:** Demographic and clinical characteristics of all included AYA cancer survivors per age‐specific care need group.

	Need (*N* = 1337)	No Need (*N* = 1644)	Do not know (*N* = 1008)	*p*‐Value
	*N* (%)	*N* (%)	*N* (%)	
Age at diagnosis (mean(SD)) in years	30.8 (6.0)	32.2 (5.7)	31.7 (5.9)	**<0.001** *
18–24 years	252 (18.8)	206 (12.5)	152 (15.1)	**<0.001**
25–34 years	628 (47.0)	706 (42.9)	443 (43.9)	
35–39 years	457 (34.2)	732 (44.5)	413 (41.0)	
Time since diagnosis (mean(SD)) in years	12.1 (4.4)	12.8 (4.5)	12.3 (4.5)	**<0.001** **
5–10 years	584 (43.7)	622 (37.8)	415 (41.2)	**0.009**
11–15 years	454 (34.0)	575 (35.0)	346 (34.3)	
16–20 years	299 (22.4)	447 (27.2)	247 (24.5)	
Sex				
Male	373 (27.9)	754 (45.9)	416 (41.3)	**<0.001**
Female	964 (72.1)	890 (54.1)	592 (58.7)	
Marital status at time of questionnaire				
Partner	1100 (82.3)	1401 (85.2)	816 (81.0)	**0.007**
No partner	234 (17.5)	235 (14.3)	187 (18.6)	
Missing	3 (0.2)	8 (0.5)	5 (0.5)	
Educational level				
No education or primary education	1 (0.1)	15 (0.9)	12 (1.2)	**<0.001**
Secondary education	62 (4.6)	128 (7.8)	73 (7.2)	
Secondary vocational education	398 (29.8)	581 (35.3)	469 (46.5)	
Higher (vocational) education	504 (37.7)	557 (33.9)	306 (30.4)	
University education	372 (27.8)	358 (21.8)	145 (14.4)	
Missing	‐	5 (0.3)	3 (0.3)	
Living status^a^				
Alone				
No	1166 (87.2)	1451 (88.3)	863 (85.6)	0.158
Yes	168 (12.6)	190 (11.6)	142 (14.1)	
Missing	3 (0.2)	3 (0.2)	3 (0.3)	
With partner				
No	424 (31.7)	426 (25.9)	319 (31.6)	**<0.001**
Yes	910 (68.1)	1215 (73.9)	686 (68.1)	
Missing	3 (0.2)	3 (0.2)	3 (0.3)	
With children				
No	565 (42.3)	757 (46.0)	431 (42.8)	0.082
Yes	769 (57.5)	884 (53.8)	574 (56.9)	
Missing	3 (0.2)	3 (0.2)	3 (0.3)	
With parents				
No	1303 (97.5)	1611 (98.0)	980 (97.2)	0.464
Yes	31 (2.3)	30 (1.8)	25 (2.5)	
Missing	3 (0.2)	3 (0.2)	3 (0.3)	
With roommates				
No	1316 (98.4)	1626 (98.9)	996 (98.8)	0.435
Yes	18 (1.3)	15 (0.9)	9 (0.9)	
Missing	3 (0.2)	3 (0.2)	3 (0.3)	
Other				
No	1333 (99.7)	1638 (99.6)	1004 (99.6)	n.a.
Yes	1 (0.1)	3 (0.2)	1 (0.1)	
Missing	3 (0.2)	3 (0.2)	3 (0.3)	
Type of cancer				
Head and neck	31 (2.3)	62 (3.8)	29 (2.9)	n.a.
Colon and rectal	27 (2.0)	27 (1.6)	28 (2.8)	
Digestive track, other	15 (1.1)	8 (0.5)	8 (0.8)	
Respiratory tract	11 (0.8)	11 (0.7)	8 (0.8)	
Melanoma	68 (5.1)	156 (9.5)	65 (6.4)	
Breast	397 (29.7)	336 (20.4)	206 (20.4)	
Female genitalia	149 (11.1)	163 (9.9)	129 (12.8)	
Male genitalia	3 (0.2)	1 (0.1)	2 (0.2)	
Urinary tract	13 (1.0)	27 (1.6)	6 (0.6)	
Thyroid gland	95 (7.1)	90 (5.5)	60 (6.0)	
Central nervous system	49 (3.7)	67 (4.1)	33 (3.3)	
Bone and soft tissue	61 (4.6)	59 (3.6)	51 (5.1)	
Germ cell tumors	166 (12.4)	355 (21.6)	170 (16.9)	
Lymphoid hematological malignancies	203 (15.2)	214 (13.0)	171 (17.0)	
Myeloid hematological malignancies	48 (3.6)	63 (3.8)	37 (3.7)	
Other	1 (0.1)	5 (0.3)	5 (0.5)	
Primary treatment^a^				
Surgery				
No	297 (22.2)	321 (19.5)	258 (25.6)	**0.001**
Yes	1038 (77.6)	1321 (80.4)	750 (74.4)	
Missing	2 (0.1)	2 (0.1)	‐	
Chemotherapy				
No	524 (39.2)	796 (48.4)	435 (43.2)	**<0.001**
Yes	811 (60.7)	846 (51.5)	573 (56.8)	
Missing	2 (0.1)	2 (0.1)	‐	
Radiotherapy				
No	646 (48.3)	932 (56.7)	518 (51.4)	**<0.001**
Yes	689 (51.5)	710 (43.2)	490 (48.6)	
Missing	2 (0.1)	2 (0.1)	‐	
Hormone therapy				
No	1110 (83.0)	1493 (90.8)	902 (89.5)	**<0.001**
Yes	225 (16.8)	149 (9.1)	106 (10.5)	
Missing	2 (0.1)	2 (0.1)	‐	
Targeted therapy				
No	1217 (91.0)	1528 (92.9)	933 (92.6)	0.146
Yes	118 (8.8)	114 (6.9)	75 (7.4)	
Missing	2 (0.1)	2 (0.1)	‐	
Stem cell therapy				
No	1293 (96.7)	1581 (96.2)	969 (96.1)	0.588
Yes	42 (3.1)	61 (3.7)	39 (3.9)	
Missing	2 (0.1)	2 (0.1)	‐	
Tumor stage				
I	523 (39.1)	774 (47.1)	417 (41.4)	**<0.001**
II	408 (30.5)	381 (23.2)	268 (26.6)	
III	189 (14.1)	223 (13.6)	159 (15.8)	
IV	61 (4.6)	69 (4.2)	49 (4.9)	
Missing	156 (11.7)	197 (12.0)	115 (11.4)	

*Note*: n.a., not applicable due to low numbers. The statistically significant values (*p* < 0.05) are in bold.

^a^ Multiple options possible.

* Post hoc test: Need vs. No need (*p* < 0.001); Need vs. Don't know (*p* = 0.001); No need vs. Don't know (*p* = 0.122)

** Post hoc test: Need vs. No need (*p* < 0.001); Need vs. Don't know (*p* = 0.467); No need vs. Don't know (*p* = 0.024).

Survivors who have had a need for age‐specific care were significantly more often female, higher educated, younger at diagnosis, and treated with chemotherapy, radiotherapy or hormone therapy, compared to those without a need and those who did not know. Those who were unsure about their need were more often without a partner and diagnosed with tumor stages III and IV, compared to those with and without a need.

### 3.2 Insight into the topics of AYAs' needs

Of those with a need for age‐specific care, 1.197 AYA cancer survivors (89.5%) provided an open text response to give further insight into their needs. All (sub) topics are described in more detail and supported by quotes to provide more insight in their meaning in Table 2.

**TABLE 2:** Quotes of AYA cancer survivors per (sub) topic.

Topic	Subtopic	Quotes
*Description*	*Description*	
Mortgages and insurances *(Life) insurance; wanting to buy a house; problems with getting a (new) mortgage; possibilities for a mortgage*		“[…] In addition, the cancer also caused us problems with getting a new mortgage. I am now (unfortunately) dependent on my husband with this as well. It would be good if information about these kinds of things was provided at an early stage, so you can at least think about the consequences. […]”
		“For example, I found out that I couldn't get a mortgage. That's pretty annoying when you're 28.”
		“Cancer and work: as a self‐employed person I could not insure myself, which shocked me.”
Friends *Peers; contact with AYA peers; social circle/life; experts by experience; reintegrating in/contributing to/participating in society; “healthy” friends; being misunderstood by peers; different life phases*;		“[…] You become far removed from the life that peers lead. They don't always understand you. That was painful. I would have liked more specific guidance on this.”
		“[…] But I did feel a gap between me and my peers. How did I deal with my new boundaries in a good way? How could I keep my confidence? How could I communicate well about this with peers? Etc…”
		“For example, being in contact with peers.”
	Peers with cancer *Peers with cancer contact; those of same age (and with similar diagnosis); advice from peers with cancer; experts by experience; exchanging experiences; acknowledgement/recognition*	“As an AYA it is hard to find peers with cancer. Older patients are in a different phase of life. Peers/friends who are not (or have not been) ill can only partially empathize with what you are going through.”
		“Absolutely, I have rarely felt so alone and couldn't find anyone in my stage of life who understood what I was going through. […]”
		“There is hardly information available with regard to my illness. I would have liked to know more about other people's experiences.”
	Social life *Social circle/life; reintegrating in/contributing to/participating in society*;	“In particular help with/information/advice about when your treatment is successful and you have been (partially) rejected by the Employee Insurance Agency (*UWV*) and sort of put aside by society, how you can still make a useful contribution to society, mentally and physically.”
		“[…] Attention for ‘reintegrating’ in society.”
		“About the impact of consequences on my social life.”
Emotions *Worried; feeling lonely/misunderstood; psychological care; confidence/self‐esteem; therapy to process everything; insecurities; fear (of recurrence/death/the future); stress; anxiety; psychologist; coping*		“After 5 years, all care is gone, but not the worries you have yourself.”
		“After the physical treatment, it still has an impact on your look at life and how you deal with the mental impact. You actually have to figure this out yourself, so it feels like you're falling into a black hole after the medical treatment.”
		“Mental support appropriate to my age would have been nice. Sometimes I felt that I had fallen between the cracks, because I was too old for the paediatric oncology ward and sometimes I felt too young to be in the ‘adult ward’. So maybe a little more tailoring.”
Appearance *Changed body; scarring; plastic surgery; breast removal*		“Due to melanoma removal next to the nipple, at that time I would have liked to be informed about the possibilities of plastic surgery to restore the shape of the breast.”
		“[…] I would also have liked to discuss the possibilities for scar correction.”
		“With reconstruction, it would be nice if there were a female doctor and nurse who could guide you through this process. In my case, it was a male doctor and I had to explain everything myself and the final result is still terrible, but the insurance no longer reimburses anything.”
Family and children *Partner; relation; (young) children; starting a/affecting family (children*, *partner); adoption; young family; parents; emotional/practical support for family; marriage/divorce*		“Insufficient attention has been paid to the family situation. There was no support regarding the situation at home during the treatments in the sense of domestic help and individual guidance. We have been left to our fate, the municipality has left all the care to the family.”
		“Attention for taking care of our, at that time, (young) children and how to explain things to them. We succeeded, but at the time there was little information about it.”
		“I would have liked more help/guidance regarding the situation at home. Young children and in the middle of a divorce.”
Sport and movement *Fitness; getting fitter/stronger; physiotherapy; exercising; sporting; guidance*		“I would have liked more aftercare because I exercised a lot and it ended abruptly. Then, the recovery was something I had to figure out myself. I would have liked some tips on how to get back to sports quickly.”
		“Did not receive physiotherapy or recovery treatment.”
		“I would have liked to exercise with supervision during my treatments.”
Late effects *Effects after 10 years*, *years after diagnosis; (checkups for) late effects; effects at older age; risks/expectations 10 years after treatment*		“You are hardly prepared for the late effects of treatment/disease.”
		“I need attention/preventative monitoring for late effects as a consequence of the treatments.”
		“In those days, it was sometimes a real search for information. The possible consequences at a later age were somewhat unclear to me at the time. […]”
Death *Disease that can kill you; dying*		“I was very young in the prime of my life and confronted with a disease from which you can die. I should have received aftercare and guidance to deal with that and to be able to give it a place. Now, 20 years have passed and it still controls my life every single day.”
		“Dealing with insecurities may be more difficult for young adults than for elderly. Besides the link between cancer and death, there are other (trivial) topics that play a role in the recovery period, such as appearance, sports, diet, etc… […]”
		“How do I tell this to my young children? How do I deal with the death sentence? What is the best way to recover? How to continue the relationship, me with a death sentence, my partner with the knowledge that she will be on her own. How to continue now that I haven't died yet, but according to the statistics I should have.”
Intimacy and sexuality *Decreased sex drive; sexologist; being intimate; sexuality*		“I had no idea that my energy and libido would be greatly reduced, as well as other side effects of my medication. Because I didn't know what ‘the standard’ was, I did not address this myself. I thought there was nothing that could be done.”
		“It might had been better to have had contact with a sexologist after the partial amputation.”
		“I had testicular cancer at the age of 22. I was treated quickly by removing the testis, but I would have liked to have gotten a prosthesis during the initial procedure. Given the age, a ‘complete’ sexual existence was desirable.”
Spirituality *(Questioning) the meaning of life; different life questions than peers*		“[…] without my partner and the support from my surroundings, for a long time, I wondered whether life still had a meaning and at the time I became seriously depressed and addicted to drugs. Experiencing problems in every imaginable part of life […] I lost the general sense of life […].”
		“[…] I had very different life questions than my peers within this group. […]”
Illness and treatment *Information about treatment*, *illness or prognosis; medical/physical care; heredity; cause of illness*;		“I was expected to make decisions about my treatment that I could not oversee at my age”
		“Now, you often think, is it because of the cancer or is it age related?”
		“Found out through a brochure that given my age, the breast cancer was something of hereditary”
	Aftercare and rehabilitation *More check‐ups; aftercare; (guided) rehabilitation; support after treatment; (more information about) recovering; physiotherapy;*	“In the year of the treatments I did not need aftercare, but years later I did. After the treatments you are ‘let go’ and you only have contact with the specialist during the check‐ups.”
		“[…] It has been more than 6 years. And maybe I am in need of extra care/guidance right now as well. The checks are always fairly tense, because how long will the medicines work? And over time I have almost developed a kind of fear of contamination for all kinds of ‘everyday’ contagious viruses etc. Because you are always afraid that it will be extra intense for yourself. This limits me sometimes or causes a lot of stress.”
		“Especially care after the medical trajectory of surgery and radiation. For the outside world you are healthy again, for yourself the processing and realization of what has happened just begins.”
	Short‐ and long‐term effects *Effect of cancer (treatment); early menopause; fatigue*, *lymphedema; side effects; lack of energy; support/information for issues/side effects*	“Especially after the treatments, when it turned out that so many adverse effects arose/remained and/or worsened. Against all my expectations and efforts.”
		“Because of the chemotherapy and later the removal of the ovaries, I entered menopause early. I would have liked some more information on this. Possibly also from peers with cancer. Complaints that I still have now often appear to be related to this, but that explanation usually only follows after I discuss the complaints.”
		“More information about fatigue after cancer and its impact on daily life.”
Work and reintegration *Work; reintegrating at work; adjusting work to one's new situation (post‐cancer); permanent or temporary contract; important to keep working*		“Particularly with regard to the reintegration process at work, which was very difficult for me and that is where you notice the gap with peers the most or what others expect based on your age.”
		“Yes, perhaps mental coaching, and job coaching to match the work well with one's physical state. Immediately after the surgery, there was help from the occupational physician for reintegration. But after that, there was no further support.”
		“What should it be like in the future when things are no longer self‐evident or possible at all due to the cancer and complications? In terms of work if you did not have a permanent contract yet and you can no longer do the work you used to do. And you fall between the cracks regarding facilities. How can you start yourself again? Who can help you with that? And that's just work…,”
Nutrition *Nutritional advice; dietician; nutrition during treatment; non‐traditional diet compared to older adults*		“Definitely. I was 33 years old when I was diagnosed with Hodgkin and would have felt supported with care to continue after cancer regarding exercise + nutritional advice + vitamin/supplements for known deficiencies after chemo and radiation.”
		“I was pregnant during the cancer. A dietitian should have been involved to make sure I ate well during chemotherapy. For me and my baby.”
		“I did not feel at home in, for example, a meeting of the hospital about healthy living after cancer, with mainly old people. I would not mind having to travel further to do something like this together with peers, I think it would be nice to exchange experiences about, for example, going to work after cancer if you are self‐employed (everything focused on at employees), how you can get back on track with your family with young children, etc. But also the practical information about nutrition. It was very focused on a traditional diet of older Dutch people. People of my generation don't eat that way at all anymore. It was of no use to me.”
Fertility and pregnancy *Wanting to have a baby/become parents; becoming pregnant; (in)fertility; pregnant and diagnosed with cancer; possible effects on fertility; fertility preservation; effect on unborn child*		“I was 31 when I was diagnosed with breast cancer. No one has asked me about my wish to have children. Everything was about making the cancer disappear. Later, partly because of the cancer treatments, I was unable to have children. I would have liked that my wish to have children was an automatic topic of conversation and perhaps something had been done with it from a medical point of view. Furthermore, there are of course more topics that should receive attention at that age, but at that time the lack of attention for my wish to have children was the most important and the most radical for me in terms of age‐specific care.”
		“We were still a young family, more attention to whether or not further expanding the family might have been nice.”
		“In my case it was not clear how much impact the chemo would have on my fertility. Later on it turned out that there were indeed risks that the doctor did not know or name. The impact on our family due to my infertility was enormous. More attention should be paid to this by informing beforehand and afterwards. There is also a life after cancer.”
Education *Continuing education; rules about study and cancer diagnosis were unknown*		“I noticed that in my surroundings at the time cancer was a much unknown subject that peers knew little about and were unaware of the seriousness. The expectations were that you would still participate in both study and external activities. This was sometimes difficult. I don't know exactly what type of support the hospital could have provided in this.”
		“I was studying at the time of my treatment and rules regarding studying were unknown to me.”
		“The doctors only have an eye for the physical complaints. I would have liked that more attention was paid to the situation I was in, namely being a student, a study that took a lot of time and energy. […]”
Benefits and compensations *Financial situation/problems/consequences; debt; income/earnings; wage discount; being declared bankrupt; financial dependence on partner*		“After my 1st diagnosis I am no longer welcome on the labor market, I have become ill a 2nd time, I fear that I have now ended up on social assistance for good. There are only a few organizations that mediate for people who have been ill.”
		“[…] Because of fatigue I had trouble standing in front of the class all day (history teacher). I quit my job and started doing other work. Eventually I got retrained and became an entrepreneur. This gave me the opportunity to manage my own time. At the same time, this has also made me more vulnerable and more financially dependent on my husband. In addition, the cancer also caused problems with us getting a new mortgage. Also in this I am now (unfortunately) dependent on my husband. It would be good if information about these things was provided at an early stage, so you can at least think about the consequences…”
		“It was very difficult for me to have cancer for so long. My peers had no idea. I often stayed with older cancer patients, who received high sickness and unemployment benefits. I did not: I got minimum. I have lived at or below the minimum for a while. When you are younger than 33, you will be compensated on minimum wage. I cannot get a mortgage, am rejected for adoption by countries, there is no understanding at the Employee Insurance Agency (*UWV*): fatigue does not exist.”
Complementary care *Mindfulness; meditation; orthomolecular doctor; massages; self‐healing ability; supplements; creative therapy*		“I think there is very little attention in the hospital for supporting you to find your own way in your recovery in other ways than just medical. I am talking about addressing the self‐healing capacity of the body. There could be more attention for that. I think there is a world to be won there in terms of mental capacity and quality of life.”
		“[…] Have had a lot of support from ‘Het behouden Huys’ (*psycho‐oncological centre*) and especially mindfulness/meditation. To better process all the sadness/uncertainty/impact. I would recommend this to anyone in the same situation. Sort of life lessons about processing/enduring traumas and rebuilding a positive self‐image and self‐confidence.”
		“During the admissions I missed massages (your body is so tired and cramped), good nutrition, creative therapy at your bed, providing more options for young people (but this may have already been done) […]”
Care not tailored *Taking into account one's age/tumor type/developmental phase; personal beliefs and physical state; intended/developed for children or older adults/elderly only; information/statistics is not tailored*		“I was being treated at the head and neck surgery department. Here you mainly see older people. Also, footage of post‐surgery scars belonged to an older man. It would be nice if there was also a younger variant for this.”
		“I didn't always feel well understood. The hospital information folder did not cover everything I was worried about…. can my relationship handle this? Does my partner still think I'm beautiful with 1 breast? Can I still exercise? How do I get back to work?”
		“I was relatively young for being diagnosed with this type of cancer. At least, that was what they always said. That made me a bit of a loner among the rest. And that made the decision making, for example about treatment, more difficult for me.”
Overarching care and life *Would have liked to receive age‐specific care; wanting to get better; lifestyle; would have liked in hindsight; diagnosis/treatment has been a long time ago; (partly) received age‐specific care; hardly any impact; unfamiliar with the term “age‐specific care”*		“At least getting the option. At this age, you may not realistically estimate the consequences.”
		“I was 37 when I was diagnosed, I did not know I belonged to the AYAs.”
		“Not at first; but when getting started after all the treatments.”
	Information and guidance *Supporting healthcare provider; support; guidance (afterwards); advice; more conversations; flyers; information; where to get information; practical help; information about limitations and possibilities*	“I really would have liked to have a little more information and clearer information.”
		“Looking back, I would have liked to receive more information about my illness and expectations. Also, there should have been more room for how best to deal with the psychological effects of the cancer at a young age.”
		“I had to do a lot of research myself, more help with this would have been very nice.”
	Life after cancer *Future; affecting one's life; effects of cancer (treatment) on life later on; rest of your life/life after cancer; expectations; general consequences in further life; daily life; quality of life*;	“This diagnosis is intense at any age, but if you are in your mid‐20s, your entire life will change. Years later, I still suffer from this.”
		“It has turned my whole future and life upside down, a lot has had to change and that is almost impossible to do.”
		“Room for what it means for the rest of your life. That you need to adjust your expectations of life and your plan for what you want and can do.”
Multiple topics per AYA		“The stage of life in combination with cancer is crucial. During the period in which I got cancer, the following things happened simultaneously: having a child, getting a mortgage, a divorce and my first permanent job.”
		“The combination of taking care of the children, physical rehabilitation, structuring work and psychological recovery was complex and I received no help, despite my request for care, which I reported to a nurse specialist, doctors and occupational physician. I was on my own. I would have liked to have already started physical rehabilitation during my treatment and learned more about cancer‐related fatigue complaints. Existing rehabilitation programs were not suitable for my situation (it was not in my place of residence, only 3 days a week, which means that it could not be combined with work reintegration, and it could only be offered as a total package, where I initially just wanted to become physically fitter).”
		“In 2006 there was little room to talk about the consequences for fertility, work and study, long‐term consequences, mortgages, etc. It took a long time to find my way and, as the only young cancer patient in my surrounding, I often felt lonely too.”
		“[…] Experiencing problems in every possible area of life. Lost my job, got rejected, suffered several burn outs, lost myself physically and mentally, lost my income, got into debt, almost lost the house, lost social contacts, lost general sense of life, became infertile.”
		“The physical treatment of the disease is as good as it can be, in our country. After 5 years, however, I have to come to the conclusion that the consequences of cancer also change all other facets of life. Such as social, relational, psychological, etc. During the physical treatment, this is briefly pointed out, but this does not stick because you are ill. After all the physical treatments there is little guidance in terms of support in these other areas. My personal need, in retrospect, is more support in these areas.”
		“I was diagnosed with cancer when my first child was 10 months old, I faced many problems caring for my child, the fear of dying and not seeing my child grow up, but also questions about the possibility of having another child after treatment.”
		“Yes, in hindsight I realize that an additional complicating factor has been my age. An age at which it is somewhat exceptional to get cancer, and in my case a recurrence. And this in an age category during which you are trying to build/shape your life, and apparently many peers cannot deal with it, which has led to a lot of abandonment in my case. I had also just quit my job and bought a new‐build home. The contract of the new job was not extended due to the recurring cancer and my house was done when I just finished treatment and therefore I immediately had to go beyond my limits.”
Overlapping topics per AYA		“Psychological care as a consequence of the removal of the uterus due to cervical cancer. Support with the psychological effect of having to accept at a ‘young’ age that you cannot have children.”
		“More attention to what cancer at a young age means for the family. Also any wish for future family expansion”
		“Not being able to work and, as a result, losing your income was especially difficult. This puts pressure on the relationship and makes it less easy to focus on one's own recovery.”
		“No psychological guidance for my husband and me and I would have liked guidance for my young children (6 and 3) during that period. I sought psychological help myself after more than 12 years….”
		“My treatment has made me go through menopause. I was barely informed about this. I had a shorter temper and did not always understand myself. I would have liked to explain this to my children.”
		“In particular more explanation regarding work and financial consequences, buying a house, etc.”
		“I have not received or been offered mental health care while to me the cancer meant that my life would be radically different than intended. Namely, I was never able to have children because of it.”
Timing of needs		“I have been in contact with peers with cancer through the AYA forum. That was pleasant, but also confrontational. The need for contact varied greatly; sometimes it was too much and my own worries/illness were enough and sometimes the need was great if I did not feel understood by my surroundings.”
		“I would have liked to be able to have group sessions/contact with peers with cancer. Maybe not immediately after diagnosis, but at a time when there was a need for it, with me that was after about six months. First on survival mode, then more processing and daring to look further.”
		“In hindsight, yes. At the time I thought I did not need it.”
Interpretation of question		“The question seems to assume that I did not receive AYA‐specific care, but I think that the care I received certainly took into account age‐specific aspects, such as fertility, follow‐up scheduling, advice on contact with peers with cancer. So I needed it, but also received it.”
		“It's hard having small children and being afraid of not seeing them grow up. In addition, I was an entrepreneur with staff. I found it very difficult to find balance, since I had less energy and time due to being sick and the treatments. I did have a lot of help though, so the question is not entirely correct: I needed help, but I also received it. So my needs have been met to a very large extent.”

**FIGURE 2:**
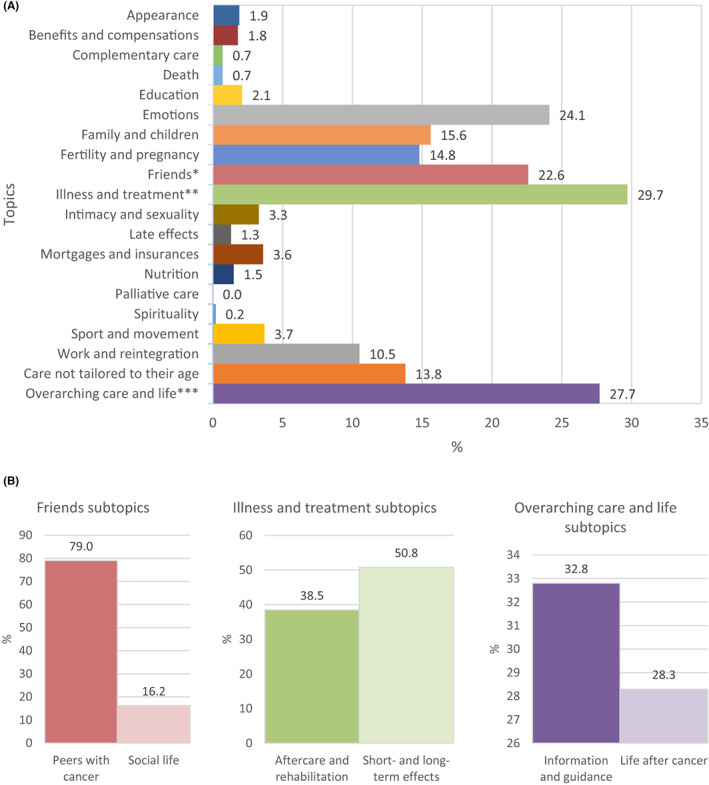
(A) The need for age‐specific care per (sub) topic. * Friends subtopics include peers with cancer and social life. ** Illness and treatment subtopics include aftercare and rehabilitation, and short‐ and long‐term effects. *** Overarching care and life subtopics include information and guidance, and life after cancer. *Note*: As some terms did not fit into one topic only, they were included into multiple topics. This was done for physiotherapy (sport and movement, and aftercare and rehabilitation) and the governmental institute for employee‐specific insurances (mortgages and insurances, work and reintegration, and benefits and compensations). Some topics are very closely related (e.g., expressing a need for psychological support for one's children and partner. Although these needs were categorized within the family and children topic only, they also link with the topic emotion). To avoid over‐categorization, these answers were categorized at one topic only. Table 2 shows several quotes as examples. An open text response can be categorized for the topic itself, in addition to the subtopic. (B) The need for age‐specific care per subtopic of friends; illness and treatment; and overarching care and life.

Most often mentioned topics of the AYA anamnesis include disease and treatment (29.7%), emotions (24.1%), friends (22.6%), family and children (15.6%), fertility and pregnancy (14.8%), and work and reintegration (10.5%) (Figure 2A and Table 2). Two subtopics emerged within the topic friends, including peers with cancer (79.0%) and social life (16.2%) (Figure 2B). A clear distinction emerged between the need for contact with peers with cancer, while on the contrary, AYAs had trouble with staying connected to their “healthy” peers (Table 2). The subtopic social life represents the society to which AYAs want to be part of (again), which goes beyond friends or family only, while simultaneously, some feel misunderstood by their surroundings. Another topic with two clear subtopics is illness and treatment, including aftercare and rehabilitation (38.5%) and short‐ and long‐term effects (50.8%) (Figure 2B). AYAs wanted information about what to expect after treatment and how to recover, as well as for check‐ups and aftercare. This is in line with the second subtopic focusing specifically on the short‐ and long‐term effects, like early menopause, fatigue or a lack of energy and lymphedema, which were often mentioned (Table 2).

Care not tailored was often mentioned explicitly (13.8%) and was therefore added as a separate topic. Examples include the experiences of AYAs that information or care was not tailored to their situation as a young cancer patient or that HCPs did not know how to communicate with them. AYAs could not identify themselves with the provided information or care: for example, it was based on older adults or they were treated as children as HCPs talked to their parents instead of to them. They had a hard time finding resources that were tailored to their specific situation, that is, age or tumor type at this young age (Table 2).

Many AYAs (27.7%) included open text responses that did not belong to any of the pre‐existing topics solely. A new topic was therefore created (overarching care and life) with two new subtopics, including information and guidance (32.8%) and life after cancer (28.3%) (Figure 2B). As with specific topics, AYAs expressed their need for more information and guidance. However, many needs are topic‐transcending and span a wider phase than solely diagnosis, treatment or follow‐up for example. In the second subtopic, a clear interest in *life after cancer* was stated, representing the restart/rest of their life, their future and everyday life (Table 2).

Least often mentioned topics include palliative care (0.0%), spirituality (0.2%), death (0.7%), complementary care (0.7%), and late effects (1.3%).

### 3.3 Additional insights of those without a need for age‐specific care or who do not know

Although the open text box was intended for those with a need for age‐specific care to specify their needs, it was also used by some of those who did not have a need (*N* = 76) or who did not know whether they have had a need (*N* = 116). Most open text responses of those without a need for age‐specific care were categorized as overarching care and life (85.5%), which was mainly about being satisfied with the received care or that the cancer has had a very minimal impact on their life. Similarly, of those who did not know whether they have had a need, 66.4% was categorized as overarching care and life. It mainly included unfamiliarity with the term “age‐specific (AYA) care,” being satisfied with the received care or that it was already a long time ago.

## 4 DISCUSSION

With almost 4000 AYA cancer survivors and a mean time from diagnosis of over 12 years, this retrospective, population‐based, observational cohort study shows that, in hindsight, many AYA cancer survivors would have loved to have received age‐specific care in the Netherlands. Most often mentioned topics include disease and treatment, work and reintegration, fertility and pregnancy, overarching care and life, care not tailored, emotions, family and children, and friends, while palliative care, complementary care, spirituality, death, and late effects were hardly mentioned. AYAs with age‐specific care needs were significantly more often female, higher educated, younger at diagnosis, and treated with chemotherapy, radiotherapy or hormone therapy. Those who have had no need or did not know if they have had a need, mainly expressed their satisfaction with the received care, the minimal impact of cancer on their lives, their unfamiliarity with the term “age‐specific (AYA) care” or that it has already been a long time ago.

Most studies so far have only focused on the first few months following diagnosis and have smaller sample sizes compared to our study.^19^, ^21^, ^22^ When survivors reflected on their experience they still remembered missing age‐specific care, some regarding multiple topics (Table 2), and some even still have unmet care needs more than 5 years after diagnosis. On average, more than one topic per person was categorized, reflecting the wide range of milestones that are so representative for this young population. The most frequently mentioned topics were in line with those from international literature.^19^, ^21^, ^22^, ^27^, ^28^, ^29^, ^30^, ^31^, ^32^ Differences in the prevalence of a topic, however, may be caused by the inclusion of different study populations (tumor types, age ranges), study designs, response options (thick boxes with provided topics or open text boxes), and (national) healthcare systems.

Both Hilgendorf et al. and Jin et al. describe the need for structured aftercare, which is in line with our results and reflected in the newly emerged subtopic aftercare and rehabilitation (Table 2).^32^, ^33^ Both the need for information and support and the need for more check‐ups after cancer treatment belong to this subtopic. Hilgendorf recommends that each AYA survivor receives a treatment summary, a survivorship care plan and education based on the AYA's age, which could help them to transition from active treatment to survivorship.^32^, ^33^

Also, in our study, many AYAs either had concerns about their fertility or they had to give up their wish of bearing a child and struggled emotionally. It was expected that this theme would be mentioned often, as it represents one of the developmental milestones which is characteristic for the AYA age range specifically (Table 2). Infertility or concerns about one's fertility are previously described in AYA literature, including potential barriers at the level of the AYA, HCP, institute and policy, as well as the effect it might have on psychological outcomes (i.e., distress, depression and health‐related quality of life).^34^, ^35^, ^36^ Although effective interventions are limited, Norton et al. describe the supporting role of nurses as a potential facilitator for fertility communication and support.^34^, ^35^

As described earlier, a new subtopic emerged based on the need for contact with peers with cancer, which is consistent with previous literature showing a clear need to connect with peers who have been diagnosed with cancer as well.^19^, ^31^, ^37^, ^38^ In the study of Zebrack et al., AYAs expressed the need of meeting other AYA cancer survivors as well and even ranked its importance higher than the support from family and friends.^31^ This might be explained by the idea that peers with cancer really understand the situation of the AYA and they can relate (i.e., similar circumstances, concerns, and interests^37^), and provide a different type of support than family and friends, which is not age‐specific per se.

The theme work and reintegration (10.5%) was more frequently mentioned than education (2.1%). This might be explained by the representation of mostly AYAs who were 25+ years of age at diagnosis, assuming that most of them finished their education and were trying to start their career/find work at time of diagnosis. However, as only few may have established career patterns, a cancer diagnosis may make it difficult to continue their work (afterwards).^39^, ^40^, ^41^ Timely involvement of an occupational health physician may support AYAs in meeting work‐related needs.^39^, ^42^

The major impact one's AYA cancer diagnosis can have on their partner, children, and family as a whole, is consistent with findings of other studies and reflected in Table 2.^21^, ^39^, ^43^, ^44^, ^45^ Many AYAs were concerned about the effect their cancer diagnosis might have on their (very young) children and had no idea how to handle this, due to the lack of support for themselves and their children. This includes both the practical support, that is, keeping the household up and running, as well as psychological support, that is, what do I tell my children, how should I do this and how will this affect them? This is in contrast with older adult cancer survivors who may actually rely on their children for practical and psychological support. Also, due to the cancer diagnosis, the AYA's partner had to take over responsibilities related to finances, work and the household, while also taking care of their sick partner. Therefore, AYAs expressed a need for support for their partner as well as for their children and both should be acknowledged as important stakeholders within the topic family and children.

A clear pattern was seen among AYAs explicitly expressing a lack of tailored care, leading to the establishment of a new subtopic called care not tailored. Information leaflets and statistics that were based on pediatric or older adult cancer survivors, HCPs who did not know how to deal with a young person and (after) care that was not in line with one's personal beliefs and physical state all belong to this subtopic. AYAs expressed a need to be seen as a young person with a certain type of cancer—being different from children and older adults—with age‐specific, individual needs. Care not tailored may in part be caused by the fact that not all HCPs and healthcare systems are aware of this unique patient population and may therefore overlook their needs. AYAs are still often considered part of the pediatric or adult cancer population instead of a distinct population: This requires an age‐specific approach integrated in the more traditional tumor oriented approach.

Topics as palliative care and death were rarely mentioned which is in line with our expectations, as the respondents are all long‐term survivors and are therefore probably less confronted with end‐of‐life issues. It was striking though that the topic late effects was only mentioned by 1.3%, as AYA cancer survivors are at increased risk of late effects, for example, subsequent malignant neoplasms, and cardiovascular diseases, compared to controls.^3^, ^46^, ^47^ It could be that terms as life after cancer, short‐/long‐term and late effects are used interchangeably, even though there are significant differences between the latter (i.e., long‐term effects can last for months or years after treatment ends, while late effects do not appear or get noticed until years after treatment ends^3^), or that AYAs are unaware of (the risks of) late effects and possibly not informed about late effects, as AYA‐specific late effects and associated risk factors are still not well understood.^46^ This is in contrast to the wealth of knowledge regarding late effects among the pediatric cancer survivor population.^48^, ^49^ As late effects may affect survivorship in terms of quantity and quality, more research on the prevalence, risk factors, and surveillance of late effects is needed. HCPs and future AYA cancer patients should be timely informed about the possible risks, symptoms, and treatment of these outcomes.

Lastly, some topics span a very wide range of issues. One of these topics is emotions, which includes, but is not limited to, loneliness, self‐esteem, stress, worrying, the need for a psychologist/therapy, coping, insecurities, and fear (of cancer recurrence/death). Although it clearly shows a need, no distinction was made between how this was expressed by participants, for example, informational needs, coaching/supervision, specific issues/effects, group therapy, resources, services, specific HCPs, or just naming the topic itself. Therefore, no specific recommendations are given on which level these needs exist, that is, the type of support needed. Future research might focus more specifically on *how* to meet the age‐specific care needs of AYAs.

### 4.1 Implications for health care and research

Based on the results, we propose to slightly adapt the framework of the current anamnesis. First, we suggest to add specific subtopics (like peers with cancer; aftercare and rehabilitation, and short‐ and long‐term effects) and more comprehensive topics (like social life including friends and family and children; and lifestyle including nutrition, sport and movement and complementary care), and to change topic names to be more inclusive (finances instead of benefits and compensations). Also, some topics are closely related, which makes them a good target for interventions. Visual rearrangements of topics, that is, putting them closely together, could show relatedness (like death close to palliative care).

If this anamnesis is implemented in the electronic patient files in the future, it might support the provision of holistic care. To use the anamnesis to its fullest potential, the AYA can read through it before one's hospital appointment and think of topics of interest to discuss. As the treating HCP might focus more on the medical side, a nurse specialist (for example focused on AYAs) can discuss the anamnesis with the AYA and make sure the patient is referred to the right specialist or intervention if needed, and stay in contact with the AYA during the disease trajectory. Using the anamnesis in the electronic patient files ensures that all multidisciplinary HCPs are up to date and aware of the AYAs' situation. In the end, this may improve awareness and communication among the involved HCPs and AYAs, help to meet the needs of AYAs and improve their outcomes. Furthermore, the results of this study can also be used to improve the AYA education module for HCPs.

The results of this study provide evidence for future research, which should assess the (added) value of AYA‐specific care, its effect on AYAs' (unmet) needs and possible gaps. In addition, as the current recommendations are based on the input of long‐term cancer survivors and needs may change over time (see Table 2 for related quotes), the input of other AYAs (based on sociodemographic and clinical data), for example those with palliative disease or with an uncertain or poor cancer prognosis,^4^ may help tailoring age‐specific care, making it more inclusive for AYAs throughout all phases of the cancer continuum. Due to the limited sample size of specific subgroups in this study, for example, AYAs who were initially diagnosed with stage IV cancer, it is difficult to draw conclusions on their needs, for which additional research is needed.

### 4.2 Strengths and limitations

This study is unique in its long‐term follow‐up and number of participants. However, it is not without limitations. Firstly, the question to assess the need for age‐specific care was not validated. Up till now, there is, however, no validated questionnaire available to assess the need for age‐specific care among AYA cancer survivors to the best of our knowledge. Also, as an open text box was used, we relied on the memory of participants to describe their needs without providing an overview of possible topics—which potentially introduced recall bias. This may have led to an underestimation of their needs, as it is quite likely that one has not remembered all their age‐specific care needs since diagnosis at the moment of questionnaire completion. However, participants have not been pushed into any direction of topics by providing predefined topics: Therefore, the topics that were mentioned must have been of real importance to them, with possibly immense (long‐term) effects, and led to the emergence of new topics. AYAs without a need or who did not know if they have had a need, expressed that they were unfamiliar with the term “age‐specific (AYA) care,” which makes it difficult to answer the question with either yes or no, and whether they received age‐specific care. Secondly, although the response rate of 35.3% is in line with previous studies, it shows that we miss the input of quite some AYAs who did not participate, which reduces generalizability.^24^ Lastly, due to the cross‐sectional design of this study, no conclusions could be drawn on possible changes in need for age‐specific care over time and per phase of the cancer continuum, including diagnosis, treatment, follow‐up, and survivorship.

## 5 CONCLUSION

To conclude, a substantial proportion of long‐term AYA cancer survivors showed a need for age‐specific care, varying by sociodemographic and clinical factors, on a wide variety of topics, which could be targeted to improve current AYA care services.

## AUTHOR CONTRIBUTIONS

**Silvie H. M. Janssen:** Conceptualization (equal); data curation (equal); formal analysis (equal); investigation (equal); methodology (equal); project administration (equal); resources (equal); software (equal); validation (equal); visualization (equal); writing – original draft (lead); writing – review and editing (lead). **Carla Vlooswijk:** Investigation (equal); writing – review and editing (supporting). **Eveliene Manten‐Horst:** Writing – review and editing (supporting). **Sophia H. E. Sleeman:** Writing – review and editing (supporting). **Rhodé M. Bijlsma:** Investigation (equal); writing – review and editing (supporting). **Suzanne E. J. Kaal:** Investigation (equal); writing – review and editing (supporting). **Jan Martijn Kerst:** Investigation (equal); writing – review and editing (supporting). **Jacqueline M. Tromp:** Investigation (equal); writing – review and editing (supporting). **Monique E. M. M. Bos:** Investigation (equal); writing – review and editing (supporting). **Tom van der Hulle:** Investigation (equal); writing – review and editing (supporting). **Roy I. Lalisang:** Investigation (equal); writing – review and editing (supporting). **Janine Nuver:** Investigation (equal); writing – review and editing (supporting). **Mathilde C. M. Kouwenhoven:** Investigation (equal); writing – review and editing (supporting). **Winette T. A. van der Graaf:** Conceptualization (equal); funding acquisition (equal); investigation (equal); methodology (equal); supervision (equal); writing – review and editing (supporting). **Olga Husson:** Conceptualization (equal); funding acquisition (equal); investigation (equal); methodology (equal); project administration (equal); supervision (equal); validation (equal); writing – review and editing (supporting).

## FUNDING INFORMATION

Dr. Olga Husson and Silvie Janssen, MSc are supported by a VIDI grant (198.007) of the Netherlands Organization for Scientific Research. Carla Vlooswijk, MSc is supported by the Dutch Cancer Society (#11788 COMPRAYA study). This research was also supported by an institutional grant of the Dutch Cancer Society and of the Dutch Ministry of Health, Welfare and Sport. Data collection of the SURVAYA study was partly supported by the investment grant (#480‐08‐009) from the Netherlands Organization for Scientific Research.

## CONFLICT OF INTEREST STATEMENT

The authors have no relevant financial or non‐financial interests to disclose.

## ETHICS STATEMENT

The study was conducted in accordance with the Declaration of Helsinki and approved by the Netherlands Cancer Institute Institutional Review Board (NCI IRB‐IRBd18122) on February 6th 2019.

## INFORMED CONSENT STATEMENT

Informed consent was obtained from all individual participants included in this study. The authors affirm that human research participants provided informed consent for publication of the quotes in Table 2.

## Data Availability

The data presented in this study are available on reasonable request from the corresponding author. The data are not publicly available due to privacy issues.

## APPENDIX 1

**TABLE A1:** Demographic and clinical characteristics of all included AYA cancer survivors in total.

	Total population (*N* = 3989)
	*N* (%)
Age at diagnosis (mean (SD)) in years	31.6 (5.9)
18–24 years	610 (15.3)
25–34 years	1777 (44.5)
35–39 years	1602 (40.2)
Time since diagnosis (mean(SD)) in years	12.4 (4.5)
5–10 years	1621 (40.6)
11–15 years	1375 (34.5)
16–20 years	993 (24.9)
Sex	
Male	1543 (38.7)
Female	2446 (61.3)
Marital status at time of questionnaire	
Partner	3317 (83.2)
No partner	656 (16.4)
Missing	16 (0.4)
Educational level	
No education or primary education	28 (0.7)
Secondary education	263 (6.6)
Secondary vocational education	1448 (36.3)
Higher (vocational) education	1367 (34.3)
University education	875 (21.9)
Missing	8 (0.2)
Living status^a^	
Alone	
No	3480 (87.2)
Yes	500 (12.5)
Missing	9 (0.2)
With partner	
No	1169 (29.3)
Yes	2811 (70.5)
Missing	9 (0.2)
With children	
No	1753 (43.9)
Yes	2227 (55.8)
Missing	9 (0.2)
With parents	
No	3894 (97.6)
Yes	86 (2.2)
Missing	9 (0.2)
With roommates	
No	3938 (98.7)
Yes	42 (1.1)
Missing	9 (0.2)
Other	
No	3975 (99.6)
Yes	5 (0.1)
Missing	9 (0.2)
Type of cancer	
Head and neck	122 (3.1)
Colon and rectal	82 (2.1)
Digestive track, other	31 (0.8)
Respiratory tract	30 (0.8)
Melanoma	289 (7.2)
Breast	939 (23.5)
Female genitalia	441 (11.1)
Male genitalia	6 (0.2)
Urinary tract	46 (1.2)
Thyroid gland	245 (6.1)
Central nervous system	149 (3.7)
Bone and soft tissue	171 (4.3)
Germ cell tumors	691 (17.3)
Lymphoid hematological malignancies	588 (14.7)
Myeloid hematological malignancies	148 (3.7)
Other	11 (0.3)
Primary treatment^a^	
Surgery	
No	876 (22.0)
Yes	3109 (77.9)
Missing	4 (0.1)
Chemotherapy	
No	1755 (44.0)
Yes	2230 (55.9)
Missing	4 (0.1)
Radiotherapy	
No	2096 (52.5)
Yes	1889 (47.4)
Missing	4 (0.1)
Hormonal therapy	
No	3505 (87.9)
Yes	480 (12.0)
Missing	4 (0.1)
Targeted therapy	
No	3678 (92.2)
Yes	307 (7.7)
Missing	4 (0.1)
Stem cell therapy	
No	3843 (96.3)
Yes	142 (3.6)
Missing	4 (0.1)
Tumor stage	
I	1714 (43.0)
II	1057 (26.5)
III	571 (14.3)
IV	179 (4.5)
Missing	468 (11.7)

^a^ Multiple options possible.
